# Preparedness for practice of newly qualified dental practitioners in the Australian context: an exploratory study

**DOI:** 10.1186/s12909-022-03684-1

**Published:** 2022-08-18

**Authors:** Rodrigo Mariño, Clare Delany, David J. Manton, Katharine Reid, Julie Satur, Felicity Crombie, Rebecca Wong, Clare McNally, Geoffrey G. Adams, Diego Lopez, Antonio Celentano, Mathew Lim, Mike Morgan

**Affiliations:** 1 Melbourne Dental School, University of Melbourne, Melbourne, Australia; 2 Melbourne Medical School, University of Melbourne, Melbourne, Australia; 3 Centre for Dentistry and Oral Hygiene, University Medical Centre Groningen, University of Groningen, Groningen, The Netherlands

**Keywords:** Oral Health, Oral Health Professionals, Preparedness to practice, Australia

## Abstract

**Background:**

The current study explored the perspectives of preparedness for dental practice from a range of relevant stakeholders (i.e., educators, employers, final-year students, graduates, practitioners, and professional associations) using an anonymous online survey in which participants described either their preparedness for practice, or the preparedness of graduates they have encountered, across six domains.

**Results:**

A total of 120 participants completed the survey. Participants were from several Australian states and territories; regional, rural, and urban locations; and working in the public and private sector. Students and new graduates generally felt prepared for activities in all the identified domains. Stakeholders reported consistently that the knowledge of dental profession graduates was at the required level to enter practice in Australia in a safe way. Activities involving the knowledge of clinical entrepreneurship and financial solvency were the dimensions where students and graduates felt least prepared (e.g., explaining fees, negotiating finances). In the domains involving clinical and technical competencies, students and new graduates self-assessed as less prepared around managing dental trauma and medical emergencies. On the other hand, activities around social and community orientation, and to a lesser extent professional attitudes and ethical judgements, were the dimensions where students and graduates felt the most prepared.

**Conclusions:**

Present findings indicate that there appear to be good standards of preparedness for practice for graduate dental professionals. This exploratory study provides insights into the nature of preparedness for Australian dental professionals and provides a basis for targeting education and professional development to address areas of need.

**Supplementary Information:**

The online version contains supplementary material available at 10.1186/s12909-022-03684-1.

## Introduction

Contemporary dental practice is more complex than at any previous time. The introduction of new procedures, technologies and treatment philosophies has created an environment that requires the ability to master complex skills and knowledge, often when there may be multiple interpretive options - referred to as ‘supercomplexity’ [1, 2]. A dental curriculum must provide the opportunity for graduates to practice safely and at an acceptable standard to ensure a high-quality health system.

Australian dental practitioners (the term ‘dental practitioner’ or ‘dental professional’ hereafter will refer to dentists, oral health therapists, dental therapists, dental hygienists, and dental prosthetists) are currently prepared for practice by thirteen higher education institutions and three Registered Training Organisations (RTOs). Individual educational providers develop their curricula in consultation with the professions and community to determine content and delivery mechanisms, informed by higher education pedagogy and a range of policy and regulatory frameworks. Higher education providers have established internal and external accreditation processes to ensure the quality and content of teaching is relevant to the needs of the community with reference to the Australian Qualifications Framework [3]. Such programs aim to produce dental practitioners who fulfil the Australian Dental Council’s (ADC) competencies of newly qualified dental practitioners in the areas of 1) Professionalism; 2) Communication and leadership; 3) Critical thinking; 4) Health promotion; 5) Scientific and clinical knowledge; and 6) Patient care (clinical information gathering; diagnosis and management planning; and clinical treatment and evaluation) [4–6].

Dental schools and training providers share a fundamental and ongoing responsibility to ensure that their graduates are entering the profession with satisfactory skills and knowledge and are safe to work as newly graduated practitioners. The ADC is particularly concerned about protection of the public and has the responsibility for accreditation of dental practitioner programs and quality assurance, thus ensuring that dental professionals have achieved the required professional competencies and are well prepared to meet Australia’s standards for practice. However, there has been minimal research exploring the extent to which dental practitioners are sufficiently prepared for practice. There is little evidence identifying the barriers to achieving workplace readiness, whether there are gaps in student preparation, or whether there is the need for more formal supports to enhance work readiness. Moreover, much of the existing research on preparation for practice is focused on the medical professions [7, 8], which have a supported one-year period of internship following initial training. Although some studies have been conducted in Australia [9–11] and overseas [12, 13]), particularly in the UK [14–17], the unique needs of dental practitioners preparing for practice as independent practitioners following graduation from their tertiary education-based training has not been considered in detail in Australia.

As part of a broader study focused on describing the key elements of preparedness for dental practice and assessing perspectives of preparedness with respect to those elements, this study aimed to explore self-reported perceptions about preparedness to practice and work readiness of newly qualified dental professional graduates in Australia [18]. Additionally, different stakeholders’ (e.g., dental course coordinators, dental educators, employers, and representatives of professional dental associations) perspectives of preparedness for practice and work readiness for Australian dental graduates were explored to determine the degree to which they corresponded with newly qualified dental professionals. This information is crucial to inform developments in educational programs for dental practitioners and ongoing professional development for newly qualified dental practitioners. It is expected that the present findings will inform future reviews and development of accreditation standards, policies, and the professional competencies of newly qualified dental practitioners.

## Methods

A group-comparison (e.g., of dental professions), cross-sectional survey research design was adopted to gather the perceptions on preparation for practice of final-year dental students, newly qualified dental professionals, and other stakeholders (e.g., employers and educators). After university ethics approval was obtained, potential participants were briefed on the aims of the study and invited to respond to the online survey delivered using Qualtrics™ (Qualtrics, UT, USA).

Perceptions of preparedness for dental practice were gathered using an instrument adapted from the Graduate Assessment of Preparedness for Practice (GAPP) questionnaire [19] with modifications to adapt to the Australian context that were informed by an initial literature review and exploratory qualitative interviews with stakeholders. The questionnaire was delivered online and comprised eight sociodemographic and work/study items and 43 items in the main questionnaire. The items in the main questionnaire were assigned to the six domains (42 items) identified by Mohan and colleagues [20]: “Academic and technical competencies”; “Communication and interprofessional skills”; “Protective mechanisms and adaptive skills”; “Professional attitude and ethical judgement”; “Clinical entrepreneurship and financial solvency”; and “Social and community orientation”. Additionally, the question: “Overall, how well-prepared for clinical practice did you feel you were when you first started practising?” was included. Respondents rated each item on a 7-point Likert score from 1 ‘Completely unprepared’ to 7 ‘Fully prepared’. New graduates and final-year dental professional students self-rated their perceived preparedness for practice and other stakeholders rated their perceptions of the preparedness of newly qualified dental graduates.

The ADC emailed an invitation to participate in the research project to groups associated with the aforementioned stakeholders to ensure the recruitment pool included all relevant practitioner groups, across all Australian states and territories, rural, regional, and metropolitan locations, and in the public and private sector. This was achieved in the following manner:

- Institutions affiliated with all accredited programs were contacted by the ADC and requested to forward the research invitation onto final-year dental students, new graduates (not more than 3 years from graduation), dental course coordinators, and dental clinical demonstrators.
- Dental professional associations (Australian Dental and Oral Health Therapists’ Association, Dental Hygienists Association of Australia, Australian Dental Association, and Australian Dental Prosthetists Association) were contacted by the ADC and requested to forward the invitation to newly graduated dental professionals, public and private-sector employers, and their members.
- The ADC invited its assessors of dental professions’ educational programs to participate in the research project.

Additionally, representatives of larger employers, both in the public and private sectors, were invited to participate.

Due to the nature of the study, there was no standardised way to calculate minimum sample size requirements for the survey component of the research. For each group of stakeholders, estimated sample sizes were established in consultation with the ADC, yielding a total estimate of 4010 potential respondents. This is equivalent to the Australia-wide total number of recent graduates and final year dental students from all five registrable dental disciplines and other stakeholders. Based on a previous study, response rates to online surveys about oral health are within the range of 2.5 to 26% [21, 22]. The project aim was to reach a 10% response rate. Thus, the calculated sample size was approximately 400 participants.

The research invitation was sent by email during March-April 2020 describing the aims of the project and including a link to access the questionnaire. The plain language statement (PLS) and participant consent form were embedded into the Qualtrics™ survey. In addition to the original research invitation, two subsequent reminder emails were sent to groups involved in recruitment.

### Data analysis

Cronbach’s alpha was used to determine the internal consistency of the instrument. The reliabilities of the six scales were found to be in the range of 0.79 to 0.96. Construct validity of the scale was assessed through a factor-analysis of the instrument’s 42 items using the maximum-likelihood estimation method with VARIMAX rotation [23]. The analysis indicated that the factor structure of GAPP scale approximated the six dimensions of the instrument, with 71.2% of the variance explained.

Comparisons between the Likert scale scores from the new graduates’ group and those from other professional groups were performed using non-parametric tests (i.e. Mann-Whitney test). A *p*-value less than 0.05 was considered statistically significant. As this was an explanatory study the *p*-values were not adjusted for multiple comparisons. All analyses were carried out using the statistical software SPSS (release 26; IBM Corporation, NY, USA).

## Results

A total of 152 new graduates/students and other stakeholders completed the online survey, thus achieving an estimated response rate of 3.8%. Thirty-two responses were excluded due to incomplete data, leaving 120 cases for full data analysis. The distribution of participants according to sociodemographic characteristics is described by reported professional group (i.e. new graduate/student or stakeholder) in Table 1. In both groups, a majority of participants were female (68.9% for new graduates and 58.1% for stakeholders). The most common year of first Australian Health Practitioner Regulation Agency (AHPRA) registration for stakeholders was prior to 2006 (48.9%) and most of the participants practiced primarily in urban settings (72.7% for new graduates and 67.3% for stakeholders).

**Table 1:** Demographic characteristics of included participants

Demographic characteristics	New Graduates / Students N* (%)	Stakeholders N* (%)
Gender		
Female	31 (68.9)	36 (58.1)
Male	14 (31.1)	24 (38.7)
Age group		
20-24	24 (57.2)	–
25-29	9 (21.4)	1 (1.6)
30-34	2 (4.8)	6 (9.7)
35-39	4 (9.5)	3 (4.8)
40-44	3 (7.1)	7 (11.3)
45-54	–	16 (25.8)
55-64	–	20 (32.3)
65+	–	9 (14.5)
Year of first registration		
< 2006	–	45 (73.8)
2006-2010	–	6 (9.8)
2011-2015	–	6 (9.8)
2016-2020	44 (100)	4 (6.6)
Primary work setting		
Urban	24 (72.7)	59 (67.3)
Regional	9 (27.3)	26 (32.7)
Primary work setting		
Private	6 (33.3)	4 (6.5)
Mix	9 (50.0)	36 (58.1)
Public	3 (16.7)	11 (17.7)
Teaching	–	6 (9.7)
Other	–	5 (8.0)
Location of education		
New South Wales	4 (8.9)	16 (25.7)
Victoria	5 (11.1)	9 (14.5)
South Australia	1 (2.2)	6 (9.7)
Western Australia	5 (11.1)	5 (8.1)
Queensland	18 (40.0)	14 (22.6)
Other or not reported	12 (26.7)	12 (19.4)

*Figures may not add due to missing values

The distribution of the age groups indicated that for new graduates/students, as expected, the majority (57.1%) were aged 20–24 years, followed by 25–29 years (21.4%). In contrast, most of the stakeholders were aged at least 45 years old (72.6%) and had 15 or more years of professional experience (73.8%). For stakeholders involved in education, the mean number of hours per week dedicated to teaching activities was 21.8 (SD 11.1; range 1-50) hours.

The distribution according to registration division by participant group is described in Table 2. Most of the participants were ADC assessors or involved in training of dental professionals (*n* = 54), followed by students (*n* = 28) and new graduates (*n* = 18). Of the 54 dental professionals were involved as either an ADC assessor or in teaching dental students; seven employers, and 13 colleagues of newly graduated dental practitioners also participated in evaluating the preparedness of new graduates. The characteristics of the clinical supervisors are presented in Tables 1 and 2.

**Table 2:** Cross-tabulation of participants groups by registration division

	Dental students	New graduates	Employer of new graduates	ADC assessor/Teaching	Other	Total
Dentist	11	9	5	24	1	50
Dental Specialist				16		16
OHT/DT	2	4	2	8	6	22
Dental Hygienist		1		2	5	8
Dental Prosthetist	3	4		3	1	11
Not specified	12			1		13
Total	28	18	7	54	13	120

*ADC* Australian Dental Council, *OHT* Oral Health Therapist, *DT* Dental Therapist

The most common dental profession was dentist (*n* = 50). Among students (*n* = 28), 39.3% were dental students and 42.9% did not specify their area of studies. Half of the new graduates were dentists and 22.2% were oral health therapists.

### Overall perception of preparedness for practice

Regarding their overall perception of preparedness for practice, more than half of all final year students (56.6%) indicated that they were ‘Undecided’, or ‘Unprepared” (Table 3). In contrast, new graduates (*n* = 14) believed that they were better prepared for practice (i.e. codes 5 or above; 85.8%) (See Table 3).

**Table 3:** Percentage frequency of students’ and new graduates’ ratings of their overall preparedness for clinical practice compared with dental professional stakeholders’ perceptions

	1 Completely unprepared	2	3	4 Undecided	5	6	7 Fully prepared
Students (*n* = 28)	4.3	17.5	8.7	26.1	26.1	13.0	4.3
New graduates (*n* = 18)	7.1	0.0	7.1	0.0	50.0	35.8	0.0
Stakeholders (*n* = 74)	0.0	7.0	8.8	7.0	49.1	26.3	1.8

When stakeholders were asked to rate how well-prepared new graduates were for clinical practice, the majority (77.1%) indicated that they were either ‘Prepared’ (49.1%), ‘Well prepared’ (26.3%), or ‘Fully prepared’ (1.8%) (Table 3). Stakeholders and new graduates rated overall preparedness similarly, with the ratings of preparedness not statistically significantly different between these groups. Interestingly, no stakeholder felt a new graduate was totally unprepared, whereas some new graduates (7.1%) believed they were.

More specifically, students’; new graduates’; and stakeholders’ perceptions of preparedness for practice according to each of the six dimensions identified by Mohan and her collaborators [20] were as follows:

### Perceptions of academic and technical competencies

There was a spread of ratings across the activities in this domain with Table 4 showing mean ratings of final-year student and new graduate self-perceived preparedness for practice on each of the academic and technical competence activities items. Overall, six activities had at least 50% of ratings greater than 5 (“Obtaining, interpreting and recording a comprehensive patient history”; “Providing relevant, comprehensive, evidence-based preventive advice to patients”; “Referring patients appropriately for advice, assessment or treatment“; “Appropriately documenting clinical findings and treatment in patient records”; “Complying with current best practice guidance in decontamination procedures and maintenance of a safe environment”; and “Showing compassion and respect to each patient and understanding the patient as a whole person rather than looking at his or her teeth in isolation” (See Table 4). Another four items; “Performing an examination and accurately identifying health, disease and abnormalities of the dentition, mouth and associated structures”; “Evaluating and monitoring the progress of treatment and dental outcomes”; “Appropriately recommending and/or administering drugs and therapeutic agents, including local anaesthesia (within the scope of practice)”; and “Possessing core scientific knowledge to support clinical practice and clinical skills necessary to provide general dental care” approached 50% (i.e. between 40.0 and 49.9%) of respondents who rated their preparedness above 5.

**Table 4:** Proportion (%) of students’ and new graduates’ level of self-reported preparedness and stakeholders’ evaluations in the academic and technical competencies domain. (*n* = 120)

	1 Completely unprepared	2	3	4 Undecided	5	6	7 Fully prepared
Obtaining, interpreting and recording a comprehensive patient history	0.0	1.9	3.7	5.6	26.2	48.6	14.0
Performing an examination and accurately identifying health, disease and abnormalities of the dentition, mouth and associated structures	0.0	1.9	5.6	9.3	40.6	38.9	3.7
Appropriately recommending and/or undertaking relevant special tests to aid diagnosis	0.9	3.7	5.6	11.1	39.8	31.5	7.4
Investigating and identifying risk factors for disease/trauma in the dentition, mouth, and associated structures	1.8	1.8	9.3	14.8	34.3	30.6	7.4
Analysing and integrating all relevant information gathered to formulate differential and definitive diagnoses	0.9	3.7	8.4	13.1	45.8	23.4	4.7
Formulating an appropriate treatment plan with the patient, taking into account the risks and benefits of treatment options	2.8	2.8	4.2	15.3	40.2	25.0	9.7
Providing relevant, comprehensive, evidence-based preventive advice to patients	0.0	0.9	6.5	11.2	28.1	36.5	16.8
Managing dental emergencies (within the scope of practice)	3.8	9.6	10.6	13.5	36.5	18.3	7.7
Identifying, assessing, and managing medical emergencies	6.1	10.1	11.1	20.2	28.2	19.2	5.1
Managing dental trauma (within the scope of practice)	3.9	15.7	10.8	20.6	27.5	18.6	2.9
Identifying, assessing, and managing pain related to the dentition, mouth and associated structures	1.9	4.6	7.4	15.7	43.5	21.3	5.6
Possessing the knowledge and skills to assess most clinical presentations	1.9	2.8	5.6	13.0	43.4	25.0	8.3
Providing required treatment to manage most clinical presentations	0.9	3.7	4.7	12.1	43.1	31.8	3.7
Using behaviour management strategies to manage patients	1.9	5.7	16.2	9.5	33.4	25.7	7.6
Evaluating and monitoring the progress of treatment and dental outcomes	2.8	3.8	9.4	9.4	33.1	31.1	10.4
Appropriately recommending and/or administering drugs and therapeutic agents, including local anaesthesia (within the scope of practice)	0.9	1.9	6.7	13.3	34.4	33.3	9.5
Being able to identify the signs of abuse or neglect in patients and raise concerns appropriately	6.3	11.6	15.9	16.8	26.3	18.9	4.2
Referring patients appropriately for advice, assessment, or treatment	1.9	2.8	4.7	9.3	30.8	38.4	12.1
Appropriately documenting clinical findings and treatment in patient records	0.0	1.9	6.6	3.8	23.6	48.1	16.0
Complying with current best practice guidance in decontamination procedures and maintenance of a safe environment	0.9	3.8	2.8	5.7	29.2	35.0	22.6
Effectively managing patients with disabilities and other special needs	3.1	14.4	11.3	18.6	29.9	19.6	3.1
Showing compassion and respect to each patient and understanding the patient as a whole person rather than looking at his or her teeth in isolation	0.9	1.9	1.9	6.7	23.8	28.6	36.2
Possessing core scientific knowledge to support clinical practice and clinical skills necessary to provide general dental care	0.0	2.9	8.6	8.6	32.4	34.3	13.2

In comparison, for two areas: “Managing dental trauma”; and “Being able to identify the signs of abuse or neglect in patients and raise concerns appropriately” overall, more than 50% of respondents rated preparedness at the midpoint (i.e., undecided) or below. The items “Identifying, assessing, and managing medical emergencies”; and “Effectively managing patients with disabilities and other special needs”; and to a lesser extent “Managing dental emergencies” approached 50% (i.e. 47.5 47.4, and 37.5%, respectively) of respondents rating preparedness at the midpoint or below (See Table 4).

Comparisons of stakeholders’ perceptions with new graduates’ self- perceptions (See supplemental Table 4a) of the academic and technical competencies of new graduates reached statistical significance, using a Mann Whitney tests, in the ratings of “Showing compassion and respect to each patient and understanding the patient as a whole person rather than looking at his or her teeth in isolation” (Mann-Whitney U = 280.5; *p* = 0.003); “Providing required treatment to manage most clinical presentations” (Mann-Whitney U = 363.0; *p* = 0.046); and ‘Analysing and integrating all relevant information gathered to formulate differential and definitive diagnoses” (Mann-Whitney U = 363.5; *p* = 0.032). Stakeholders generally scored new graduates lower, but still towards the prepared side (rating > 4), as compared to the self-assessment by new graduates.

Comparisons of students and new graduates’ ratings differed significantly in this domain for three items (*p* < 0.05). In these three cases (“Managing dental emergencies” (Mann-Whitney U = 124.0; *p* = 0.011); “Analysing and integrating all relevant information gathered to formulate differential and definitive diagnoses” (Mann-Whitney U = 118.0; *p* = 0.006); and “Providing required treatment to manage most clinical presentations”; (Mann-Whitney U = 147.0; *p* = 0.043), new graduates tended to rate themselves as more prepared for practice compared to final year students (See supplemental Table 4a).

### Perceptions of communication and interprofessional skills

In two of the four activities in the communication and interprofessional skills domain for students and new graduates, more than 50% of ratings in both groups were greater than 5 on the 7-point scale (Table 5). In the remaining activities: “Discussing sensitive issues with patients and caregivers, negotiating payment options, and communicating effectively in a professional team”; and “Communicating feedback appropriately with colleagues from dental and other healthcare professions, and raising concerns when problems arise”, participants rated at the midpoint of the scale (i.e. undecided) or below (43.0 and 35.0%, respectively).

**Table 5:** Proportion (%) of students’ and new graduates’ level of self-reported preparedness and stakeholders’ evaluations in the communication and interprofessional skills domain. (*n* = 120)

	1 Completely unprepared	2	3	4 Undecided	5	6	7 Fully prepared
Discussing diagnosis and treatment plans effectively, explaining the benefits, risks and discomfort related to treatment, preventive health strategies and post-operative instructions*	1.0	1.9	7.7	10.6	27.9	41.3	9.6
Communicating appropriately, effectively and sensitively at all times with and about patients, their representatives and the general public, and obtaining informed consent	1.0	1.9	4.8	7.7	31.7	32.7	20.2
Discussing sensitive issues with patients and caregivers, negotiating payment options and communicating effectively in a professional team	0.0	14.0	9.0	20.0	26.0	22.0	9.0
Communicating feedback appropriately with colleagues from dental and other healthcare professions, and raising concerns when problems arise	0.0	4.9	8.7	21.4	35.0	19.3	10.7

Comparisons of stakeholders’ perceptions with new graduates’ self- perceptions reached statistically significant differences in “Discussing diagnosis and treatment plans effectively, explaining the benefits, risks and discomfort related to treatment, preventive health strategies and post-operative instructions”. Stakeholders scored new graduates lower, but still towards the prepared side, compared to new graduates themselves (Mann-Whitney U = 320.0; *p* = 0.027). No statistically significant differences between students and new graduates were evident in any of the activities in this domain (See Table supplemental 5a).

### Perceptions of protective mechanisms and adaptive skills

In all the activities of this domain at least 55% of participants rated 5 or above (Table 6). There were no statistically significant differences between students and graduates or between graduates and stakeholders’ ratings in this domain (Table 6).

**Table 6:** Proportion (%) of students’ and new graduates’ level of self-reported preparedness and stakeholders’ evaluations in the protective mechanisms and adaptive skills domain. (*n* = 120)

	1 Completely unprepared	2	3	4 Undecided	5	6	7 Fully prepared
Understanding the importance of keeping up to date and committing to lifelong learning, understanding the importance of reflective learning, feedback and development*	0.0	2.9	4.8	8.6	21.0	30.5	32.4
Evaluating clinical research and evidence and adapting to relevant, emerging and new technology and techniques	0.0	6.8	4.9	18.4	29.1	26.2	14.6
Being able to cope with diverse work situations, managing time, coping with stress and effectively balancing work and personal life	1.0	10.0	10.0	21.0	31.0	15.0	12.0

### Perceptions of professional attitude and ethical judgement

With one exception, in all activities of this domain, at least 50% of participants rated 5 or above (Table 7). The exception was “Leading, managing and taking professional responsibility for the actions of colleagues and other members of the team involved in patient care”, where at least 70% of participants rated new graduates/students at, or above, the midpoint of the scale.

**Table 7:** Proportion (%) of students’ and new graduates’ level of self-reported preparedness and stakeholders’ evaluations in the professional attitude and ethical judgement. (*n* = 120)

	1 Completely unprepared	2	3	4 Undecided	5	6	7 Fully prepared
	Students and new graduates%/Stakeholders%						
Respecting patients’ dignity and choices and providing care according to the patient’s needs and culture*	0.0	2.0	2.9	10.8	22.5	37.3	24.5
Recognising and acting within the Dental Board of Australia’s standards and within other professionally relevant laws, ethical guidance and systems	0.0	2.0	2.9	14.7	19.6	33.3	27.5
Understanding the roles of, and cooperating effectively with, other members of the healthcare team in the best interests of patients*	0.0	2.2	6.5	10.8	24.7	31.1	24.7
Recognising the importance of and demonstrating personal accountability to patients, the regulator, the team and wider community, and putting patients’ interests first and acting as their advocate where appropriate*	0.0	1.1	5.4	15.1	22.6	39.7	16.1
Leading, managing and taking professional responsibility for the actions of colleagues and other members of the team involved in patient care***	1.1	4.6	4.6	17.2	32.3	27.6	12.6
Recognising and complying with local and national systems and processes to support safe patient care, including the safe use of equipment and materials	0.0	2.1	5.3	14.9	26.6	33.0	18.1

Students and new graduates did not differ statistically on how they rated the various professional attitudes and ethical judgement domain activities. However, statistically significant differences were present in two of the six activities regarding stakeholders’ assessments responses as compared with that of the new graduates/students: “Understanding the roles of, and cooperating effectively with, other members of the healthcare team in the best interest of patients” (Mann-Whitney U = 198.5; *p* = 0.015) (See Table supplemental 7a); and “Leading, managing and taking professional responsibility for the actions of colleagues and other members of the team involved in patient care” (Mann-Whitney U = 134.0; *p* < 0.001).

### Perceptions of clinical entrepreneurship and financial solvency

Students and new graduates rated themselves on these items as less prepared in all three areas of the clinical entrepreneurship and financial solvency domain (Table 8), with the majority (70% or greater) scoring below 6. There were no statistically significant differences between students and new graduates’ perceptions in any of the activities in this domain.

**Table 8:** Proportion (%) of students’ and new graduates’ level of self-reported preparedness and stakeholders’ evaluations in the clinical entrepreneurship and financial solvency domain (*n* = 120)

	1 Completely unprepared	2	3	4 Undecided	5	6	7 Fully prepared
	Students and new graduates%/Stakeholders%						
Respecting patients’ dignity and choices and providing care according to the patient’s needs and culture*	0.0	2.0	2.9	**10.8**	22.5	37.3	24.5
Recognising and acting within the Dental Board of Australia’s standards and within other professionally relevant laws, ethical guidance and systems	0.0	2.0	2.9	14.7	19.6	33.3	27.5
Understanding the roles of, and cooperating effectively with, other members of the healthcare team in the best interests of patients*	0.0	2.2	6.5	10.8	24.7	31.1	24.7
Recognising the importance of and demonstrating personal accountability to patients, the regulator, the team and wider community, and putting patients’ interests first and acting as their advocate where appropriate*	0.0	**1.1**	5.4	15.1	22.6	39.7	16.1
Leading, managing and taking professional responsibility for the actions of colleagues and other members of the team involved in patient care***	1**.1**	4.6	4.6	17.2	32.3	27.6	12.6
Recognising and complying with local and national systems and processes to support safe patient care, including the safe use of equipment and materials	0.0	2.1	5.3	14.9	26.6	33.0	18.1

When comparing new graduates’ ratings with those of the stakeholders, there was a statistically significant difference in the rating of “Understanding the interface between clinical practice, patient care, and operating a business in conjunction with one’s professional and legal obligations as a health professional” in that graduate rated themselves as more prepared than stakeholders did (U = 211.0; *p* = 0.033). There were no statistically significant differences between new graduates and stakeholders’ perceptions for the other two activities in this domain (See supplemental Table 8a).

### Perceptions of social and community orientation

Across the activities in the social and community orientation domain, at least 60% of respondents’ ratings were greater than 5 on the 7-point scale (Table 9). There were no statistically significant differences between either stakeholders and new graduates or between students’ and new graduates’ perceptions for any of the activities in this domain (See supplemental Table 9a).

**Table 9:** Proportion (%) of students’ and new graduates’ level of self-reported preparedness and stakeholders’ evaluations in the social and community orientation domain (*n* = 120)

	1 Completely unprepared	2	3	4 Undecided	5	6	7 Fully prepared
Providing culturally safe care that recognises the distinct needs of Aboriginal and Torres Strait Islander Peoples in relation to dental care provision	3.5	5.9	7.1	20.0	24.7	25.9	12.9
Understanding the current issues relating to inequalities in dental, and how to plan to address these needs	0.0	6.8	9.1	22.7	23.9	23.9	13.6
Evaluating the impact of social factors on illness, holistically understanding the social situations of one’s patients and their families and/or carers*	0.0	4.3	6.5	20.7	33.8	21.7	13.0

## Discussion

In this research, the assessment of preparedness for practice based on self-perceptions and experiences of final-year dental professional students and new graduates was contrasted with the perspectives of dental professional stakeholders including tutors, educational supervisors, and senior members of the profession. Key findings demonstrated that although there was some spread of ratings over the 42 activities, most respondents in the study indicated that newly qualified dental professionals were, by and large, prepared to practice safely. There was also general agreement from the views of stakeholders, that newly graduated dental professionals are entering the health care system with acceptable levels of clinical skills and no specific clinical areas or procedures were identified where preparedness is of concern.

A notable finding was that new graduates self-perceived that they were more prepared for practice compared with dental professional stakeholders’ perceptions of their preparedness. In seven of the 42 comparisons, differences reached the level of statistical significance. Additionally, as expected, graduates with some post-graduation clinical experience believed that they were more prepared for practice than final year students. However, it must be acknowledged that the students were surveyed at the beginning of their final year, with some months of additional training ahead before they graduated. Their perspectives may have been different just after the end of teaching. Additionally, this pattern acknowledges that in the acquisition and development of a skill, a dental professional progresses through different levels of proficiency: from novice or advanced beginner, towards expertise, with increasing exposure to work and additional experience of engagement in dental practice [24]. Thus, it is expected that students will not have the same confidence, compared to new graduates, to be able to complete clinical tasks independently, and therefore feel less prepared.

Communication skills were perceived as one of the strongest areas of preparedness for practice across all respondents. Both stakeholders and new graduates judged that preparedness was high for communication skills. This included skills in interactional and interpersonal skills with patients and colleagues as well as interprofessional communication [25, 26]. This is encouraging as communication skills have been placed among the most important traits of a good oral health professional [4–6, 16], and one for which new graduates have previously reported the need for more coverage [17, 26–28]. However, consistent with the literature, new graduates still felt less prepared to discuss sensitive issues with patients and caregivers [25].

The highest ratings of self-perceived preparedness were also given to aspects such as professional attitude, ethical judgement and social and community orientation (i.e., showing compassion and respect to patients). This was followed by an item in the protective mechanisms and adaptive skills dimension (e.g., committing to lifelong learning). Interestingly, students and new graduates generally indicated that they were unprepared for coping with stressors (i.e., anxiety and patient expectations), for the business aspects of private dental practice and for the complexity of managing a private dental practice.

To some extent, in the domains involving clinical and technical competencies, and clinical entrepreneurship, new graduates self-assessed as less prepared for practice. These findings are similar to studies and other discussions in the literature [25, 28, 29]. For example, for clinical competencies, students, and new graduates self-assessed as less prepared for managing dental trauma and identifying, assessing, and managing medical emergencies. It was acknowledged that the organisation of the course components might give fewer opportunities for learning about these skills with patients [30]. Data from new graduates and stakeholders highlighted the importance of the real-world experience of the clinical environments [30]. For example, providing treatment away from general hospital settings may give fewer opportunities for a more holistic view of patient care, beyond their oral health.

Activities involving clinical entrepreneurship and financial solvency were dimensions of practice where students and graduates felt least prepared (e.g., to explain fees, or negotiate finances) compared to the six other dimensions. These findings are largely consistent with the literature for domains in which graduates felt more insecure [26, 27, 31–33]. However, to contextualise these results in Australian settings, although some dental training providers operate their own clinics (e.g., Oral Health Centre of Western Australia; Melbourne Dental Clinic), most dental training in Australia involves providing care to public patients where treatment is fully subsidised. Therefore, there is limited exposure to private dentistry or a chance to gain experience in these educational contexts. This is an area of preparedness which most new graduates might need to develop when they start practising, particularly in the private sector.

There is also a need to highlight the distinction between ‘work-readiness’ and ‘clinical competence/proficiency’. Only one of the six domains of work readiness that we used in the survey [20] specifically refers to a competency (Academic and technical competencies). Each of the other domains (‘Communication and interprofessional skills’; ‘Protective mechanisms and adaptive skills’; ‘Professional attitude and ethical judgement’; ‘Clinical entrepreneurship and financial solvency’; and ‘Social and community orientation’) references broader skills and characteristics required for negotiating different workplace contexts and building collegial and patient relationships. This distinction between competency and preparedness for practice is important and was the underlying premise for this research. It highlights the importance of broadening the educational focus of dental practitioners beyond attaining technical skills.

The findings need to be considered and interpreted with a degree of caution. For example, relative to the size of the target population, the survey sample for all participant groups was limited, although within the range for online surveys about oral health [21, 22]. Therefore, the trends presented here should be considered as exploratory and designed to stimulate debate, discussion and further research. The final sample, however, achieved a representation of all dental professions and most dental schools, and fulfilled minimum requirements for quantitative data analysis. The timing of the study, with That the invitation to participate sent in late February 2020, just prior to the COVID-19 pandemic lockdown in many parts of Australia, may have contributed to the low response rate [34, 35]. Another limitation was the self-reported nature of the responses, that may have either overstated or understated preparedness for practice assessments.

Nonetheless, despite these limitations, the similarities between these exploratory quantitative findings and the associated qualitative study [30] would indicate that it may well reflect the true level of preparedness for practice of new dental graduates. As such, this research adds considerable evidence for the identification of areas in need of improvement, as well as those areas achieving good standards. Additionally, the present study describes, for the first time, Australian newly graduated dental professionals’ preparedness for clinical practice.

The results highlight that despite different curricula, approaches to teaching and learning, and methods of student intake (i.e., undergraduate and graduate entry) of the dental schools represented in this study, preparedness was assessed as high across the sample. Furthermore, the assessment of preparedness from new graduates and from dental educators and clinical supervisors was consistent across these groups. Any statistically significant differences were more in the strength of the response, rather than the direction, which generally reflected preparedness.

Stakeholders’ perceptions of preparedness may have been influenced by profession-specific concepts around preparedness. Thus, stakeholders’ assessments might have been guided by their personal concept of how dentistry should be practiced, which may not be a universally held view across the dental professions [30, 36]. Preparedness for practice means that the new graduate is ready to function independently in a diverse range of environments, not only being competent for just clinical practice [20]. New graduates may not be prepared for the business aspect of private dental practice. However, there were generally few concerns about the clinical skills of graduates, except for a few specific scenarios (managing dental trauma and identifying, assessing, and managing medical emergencies).

## Conclusions

At the time of graduation, new graduates are expected to have achieved certain competencies to enable them to practise independently. The present findings indicate that Australian dental students appear to be acquiring adequate theoretical and evidence-based information in their formal learning and teaching activities, which prepares them to practice as dental practitioners. Notwithstanding this, specific areas were identified in which new graduates and students may benefit from further training and consolidation, as well as areas where higher levels of experience might be required. Nevertheless, consistent with the literature, it is generally acknowledged that consolidating competencies in clinical practice is a lifelong learning process [15]. The present data highlights areas in dental education that could be strengthened, as well as areas considered to be achieving good standards. The findings point to the potential learning benefit for graduates of active involvement and exposure to real-life clinical situations including more exposure to the business/financial aspects of practice, involvement in general dental practice, and interacting with other health professionals.

## Supplementary Information

**Additional file 1: Table 4a.** Proportion (%) of students’ and new graduates’ level of self-reported preparedness and stakeholders’ evaluations in the academic and technical competencies domain*.

**Additional file 2: Table 5a.** Proportion (%) of students’ and new graduates’ level of self-reported preparedness and stakeholders’ evaluations in the communication and interprofessional skills domain.*

**Additional file 3: Table 6a.** Proportion (%) of students’ and new graduates’ level of self-reported preparedness and stakeholders’ evaluations in the protective mechanisms and adaptive skills domain.*

**Additional file 4: Table 7a.** Proportion (%) of students’ and new graduates’ level of self-reported preparedness and stakeholders’ evaluations in the professional attitude and ethical judgement.

**Additional file 5: Table 8a.** Proportion (%) of students’ and new graduates’ level of self-reported preparedness and stakeholders’ evaluations in the clinical entrepreneurship and financial solvency domain.*

**Additional file 6: Table 9a.** Proportion (%) of students’ and new graduates’ level of self-reported preparedness and stakeholders’ evaluations in the social and community orientation domain.*

## Data Availability

The datasets generated and/or analysed during the current study are not publicly available due to the ethics approval granted on the basis that only researchers involved in the study can access the de-identified data. The minimum retention period is five years from publication. Supporting documents are available upon request to the corresponding author.

## Abbreviations

ADC: Australian Dental Council
GAPP: Graduate Assessment of Preparedness for Practice
