# Supplementary material for: Preparedness for practice of newly qualified dental practitioners in the Australian context: an exploratory study
Source: BMC Med Educ. 2022 Aug 18;22:625. doi: 10.1186/s12909-022-03684-1 (PMC9385413; doi:10.1186/s12909-022-03684-1)
Supplement: Supplementary file 1 — Additional file 1: Table 4a. Proportion (%) of students’ and new graduates’ level of self-reported preparedness and stakeholders’ evaluations in the academic and technical competencies domain*. [file 12909_2022_3684_MOESM1_ESM.docx]

Table 4a. Proportion (%) of students’ and new graduates’ level of self-reported preparedness and stakeholders’ evaluations in the academic and technical competencies domain*

|  | 1  Completely  unprepared | 2 | 3 | 4  Undecided | 5 | 6 | 7  Fully prepared |
| --- | --- | --- | --- | --- | --- | --- | --- |
|  | Students%/New graduates%**/Stakeholders**% | | | | | | |
| Obtaining, interpreting and recording a comprehensive patient history | 0.0/0.0/**0.0** | 0.0/5.6/**1.7** | 3.8/0.0/**5.2** | 11.5/5.6/**1.7** | 30.5/16.7/**24.1** | 38.8/44.3/**58.7** | 15.4/27.8/**8.6** |
| Performing an examination and accurately identifying health, disease and abnormalities of the dentition, mouth and associated structures | 0.0/0.0/**0.0** | 0.0/0.0/**3.4** | 11.5/11.1/**1.7** | 15.5/5.6/**6.8** | 53.8/27.8/**40.6** | 11.5/55.5/**44.1** | 7.7/0.0/**3.4** |
| Appropriately recommending and/or undertaking relevant special tests to aid diagnosis | 0.0/5.6/**0.0** | 0.0/5.6/**5.1** | 3.8/5.6/**5.1** | 11.5/5.6/**11.9** | 42.4/22.2/**42.3** | 26.9/49.8/**30.5** | 15.4/5.6/**5.1** |
| Investigating and identifying risk factors for disease/trauma in the dentition, mouth, and associated structures | 0.0/0.0/**3.4** | 0.0/5.6/**1.7** | 19.2/11.1/**3.4** | 19.2/5.6/**16.9** | 34.6/27.8/**33.2** | 11.6/44.3/**36.3** | 15.4/5.6/**5.1** |
| Analysing and integrating all relevant information gathered to formulate differential and definitive diagnoses | 0.0/5.6/**0.0** | 0.0/0.0/**6.8** | 12.0/0.0/**8.5** | 32.0/5.6/**8.5** | 40.0/33.3/**49.1** | 12.0/44.4/**23.7** | 4.0/11.1/**3.4** |
| Formulating an appropriate treatment plan with the patient, taking into account the risks and benefits of treatment options | 0.0/0.0/**3.7** | 0.0/5.6/**3.7** | 8.0/0.0/**3.7** | 32.0/5.6/**7.4** | 28.0/44.4/**51.9** | 28.0/22.2/**22.2** | 4.0/22.2/**7.4** |
| Providing relevant, comprehensive, evidence-based preventive advice to patients | 0.0/0.0/**0.0** | 0.0/0.0/**1.7** | 7.7/5.6/**5.2** | 15.4/0.0/**12.1** | 23.1/22.2/**32.7** | 34.6/44.4/**36.2** | 19.2/27.8/**12.1** |
| Managing dental emergencies (within the scope of practice) | 8.0/5.6/**1.8** | 16.0/4.0/**10.7** | 16.0/5.6/**7.1** | 20.0/11.2/**12.5** | 24.0/33.3/**44.6** | 12.0/33.3/**14.4** | 4.0/7.0/**8.9** |
| Identifying, assessing, and managing medical emergencies | 7.7/11.1/**4.0** | 23.1/5.6/**6.0** | 7.7/5.6/**14.0** | 26.9/11.1/**18.0** | 15.4/27.7/**36.0** | 15.4/33.3/**16.0** | 3.8/5.6/**6.0** |
| Managing dental trauma (within the scope of practice) | 11.6/5.6/**0.0** | 15.4/22.2/**13.2** | 11.5/0.0/**13.2** | 30.8/16.7/**17.0** | 23.1/22.2/**33.9** | 3.8/33.3/**18.9** | 3.8/0.0/**3.8** |
| Identifying, assessing, and managing pain related to the dentition, mouth and associated structures | 7.7/0.0/**0.0** | 0.0/5.6/**6.8** | 7.7/11.1/**5.1** | 26.9/16.7/**13.6** | 34.7/44.3/**47.4** | 19.2/11.1/**22.0** | 3.8/11.1/**5.1** |
| Possessing the knowledge and skills to assess most clinical presentations | 3.8/5.6/**0.0** | 0.0/0.0/**5.1** | 3.8/0.0/**6.8** | 15.4/11.1/**11.9** | 50.0/33.3/**44.1** | 23.2/27.8/**25.3** | 3.8/22.2/**6.8** |
| Providing required treatment to manage most clinical presentations | 4.0/0.0/**0.0** | 8.0/0.0/**3.4** | 4.0/0.0/**6.8** | 12.0/11.1/**11.8** | 44.0/33.3/**44.1** | 24.0/44.5/**32.2** | 4.0/11.1/**1.7** |
| Using behaviour management strategies to manage patients | 4.0/0.0/**1.8** | 4.0/5.6/**7.0** | 16.0/11.1/**15.8** | 8.0/5.6/**8.8** | 36.0/22.2/**36.7** | 24.0/38.8/**24.6** | 8.0/16.7/**5.3** |
| Evaluating and monitoring the progress of treatment and dental outcomes | 4.0/0.0/**3.4** | 4.0/5.6/**3.4** | 4.0/0.0/**13.8** | 16.0/11.1/**5.2** | 28.0/33.3/**34.5** | 28.0/27.8/**34.5** | 16.0/22.2/**5.2** |
| Appropriately recommending and/or administering drugs and therapeutic agents, including local anaesthesia (within the scope of practice) | 00/5.6/**0.0** | 0.0/0.0/**3.5** | 8.0/0.0/**7.0** | 24.0/0.0/**12.3** | 36.0/38.8/**33.3** | 20.0/33.3/**38.6** | 12.0/22.2/**5.3** |
| Being able to identify the signs of abuse or neglect in patients and raise concerns appropriately | 8.0/11.1/**4.2** | 16.0/5.6/**12.5** | 20.0/16.7/**14.6** | 12.0/5.6/**25.0** | 32.0/22.2/**18.8** | 4.0/33.3/**22.8** | 8.0/5.6/**2.1** |
| Referring patients appropriately for advice, assessment, or treatment | 4.0/5.6/**0.0** | 0.0/5.6/**3.4** | 4.0/5.6/**5.1** | 12.0/5.6/**8.5** | 52.0/16.7/**25.1** | 20.0/38.7/**44.1** | 8.0/22.2/**13.8** |
| Appropriately documenting clinical findings and treatment in patient records | 0.0/0.0/**0.0** | 0.0/5.6/**1.7** | 8.0/0.0/**6.9** | 8.0/0.0/**3.4** | 32.0/11.1/**24.1** | 28.0/61.1/**51.8** | 24.0/22.2/**12.1** |
| Complying with current best practice guidance in decontamination procedures and maintenance of a safe environment | 00/5.6/**0.0** | 4.0/0.0/**5.2** | 0.0/5.6/**1.7** | 4.0/5.6/**3.4** | 32.0/33.3/**29.3** | 20.0/16.6/**46.6** | 40.0/33.3/**13.8** |
| Effectively managing patients with disabilities and other special needs | 8.0/5.6/**0.0** | 12.0/22.2/**14.0** | 20.0/0.0/**12.0** | 8.0/11.1/**24.0** | 40.0/22.2/**26.0** | 12.0/27.8/**22.0** | 0.0/11.1/**2.0** |
| Showing compassion and respect to each patient and understanding the patient as a whole person rather than looking at his or her teeth in isolation | 0.0/5.6/**0.0** | 0.0/0.0/**3.5** | 0.0/0.0/**3.5** | 4.0/0.0/**8.8** | 12.0/11.1/**29.8** | 20.0/22.2/**35.1** | 64.0/61.1/**19.3** |
| Possessing core scientific knowledge to support clinical practice and clinical skills necessary to provide general dental care | 0.0/0.0/**0.0** | 4.0/0.0/**3.4** | 12.0/5.9/**6.9** | 20.0/5.9/**3.4** | 32.0/29.4/**32.8** | 16.0/35.3/**43.2** | 16.0/23.5/**10.3** |

* Students (n=28); New graduates (n=18); Stakeholders (n=74)
