# Supplementary material for: Preparedness for practice of newly qualified dental practitioners in the Australian context: an exploratory study
Source: BMC Med Educ. 2022 Aug 18;22:625. doi: 10.1186/s12909-022-03684-1 (PMC9385413; doi:10.1186/s12909-022-03684-1)
Supplement: Supplementary file 2 — Additional file 2: Table 5a. Proportion (%) of students’ and new graduates’ level of self-reported preparedness and stakeholders’ evaluations in the communication and interprofessional skills domain.* [file 12909_2022_3684_MOESM2_ESM.docx]

Table 5a. Proportion (%) of students’ and new graduates’ level of self-reported preparedness and stakeholders’ evaluations in the communication and interprofessional skills domain.*

|  | 1  Completely  unprepared | 2 | 3 | 4  Undecided | 5 | 6 | 7  Fully prepared |
| --- | --- | --- | --- | --- | --- | --- | --- |
|  | Students%/New graduates%**/Stakeholders**% | | | | | | |
| Discussing diagnosis and treatment plans effectively, explaining the benefits, risks and discomfort related to treatment, preventive health strategies and post-operative instructions | 0.0/0.0/**1.8** | 4.0/0.0/**1.8** | 4.0/0.0/**10.5** | 12.0/5.9/**10.5** | 16.0/29.4/**31.6** | 52.0/41.2/**38.5** | 12.0/23.5/**5.3** |
| Communicating appropriately, effectively and sensitively at all times with and about patients, their representatives and the general public, and obtaining informed consent | 0.0/5.9/**0.0** | 4.0/0.0/**1.8** | 4.0/0.0/**7.0** | 12.0/0.0/**8.8** | 16.0/29.4/**35.1** | 32.0/41.2/**31.5** | 32.0/23.5/**15.8** |
| Discussing sensitive issues with patients and caregivers, negotiating payment options and communicating effectively in a professional team | 0.0/0.0/**0.0** | 16.0/5.9/**16.6** | 8.0/5.9/**11.1** | 16.0/29.4/**16.7** | 28.0/23.5/**24.1** | 20.0/23.5/**24.1** | 12.0/11.8/**7.4** |
| Communicating feedback appropriately with colleagues from dental and other healthcare professions, and raising concerns when problems arise | 0.0/0.0/**0.0** | 4.0/5.9/**5.4** | 12.0/5.9/**8.9** | 28.0/17.6/**19.6** | 36.0/35.4/**33.9** | 8.0/17.6/**23.3** | 12.0/17.6/**8.9** |

* Students (n=28); New graduates (n=18); Stakeholders (n=74)
