# Supplementary material for: Preparedness for practice of newly qualified dental practitioners in the Australian context: an exploratory study
Source: BMC Med Educ. 2022 Aug 18;22:625. doi: 10.1186/s12909-022-03684-1 (PMC9385413; doi:10.1186/s12909-022-03684-1)
Supplement: Supplementary file 3 — Additional file 3: Table 6a. Proportion (%) of students’ and new graduates’ level of self-reported preparedness and stakeholders’ evaluations in the protective mechanisms and adaptive skills domain.* [file 12909_2022_3684_MOESM3_ESM.docx]

Table 6. Proportion (%) of students’ and new graduates’ level of self-reported preparedness and stakeholders’ evaluations in the protective mechanisms and adaptive skills domain.*

|  | 1  Completely  unprepared | 2 | 3 | 4  Undecided | 5 | 6 | 7  Fully prepared |
| --- | --- | --- | --- | --- | --- | --- | --- |
|  | Students%/New graduates%**/Stakeholders**% | | | | | | |
| Understanding the importance of keeping up to date and committing to lifelong learning, understanding the importance of reflective learning, feedback and development* | 0.0/0.0/**0.0** | 4.0/0.0/**3.4** | 0.0/5.9/**5.2** | 8.0/0.0/**10.3** | 12.0/35.3/**22.4** | 16.0/29.4/**34.5** | 60.0/29.4/**24.2** |
| Evaluating clinical research and evidence and adapting to relevant, emerging and new technology and techniques | 0.0/0.0/**0.0** | 8.0/11.8/**5.4** | 4.0/0.0/**5.**4 | 16.0/11.8/**21.4** | 24.0/47.0/**25.0** | 24.0/11.8/**32.1** | 24.0/17.6/**10.7** |
| Being able to cope with diverse work situations, managing time, coping with stress and effectively balancing work and personal life | 4.0/0.0/**0.0** | 12.0/5.9/**11.1** | 4.0/5.9/**13.0** | 28.0/11.8/**18.5** | 20.0/35.3/**33.3** | 16.0/23.5/**16.7** | 16.0/5.9/**7.4** |

* Students (n=28); New graduates (n=18); Stakeholders (n=74)
