# Supplementary material for: Preparedness for practice of newly qualified dental practitioners in the Australian context: an exploratory study
Source: BMC Med Educ. 2022 Aug 18;22:625. doi: 10.1186/s12909-022-03684-1 (PMC9385413; doi:10.1186/s12909-022-03684-1)
Supplement: Supplementary file 4 — Additional file 4: Table 7a. Proportion (%) of students’ and new graduates’ level of self-reported preparedness and stakeholders’ evaluations in the professional attitude and ethical judgement. [file 12909_2022_3684_MOESM4_ESM.docx]

Table 7a. Proportion (%) of students’ and new graduates’ level of self-reported preparedness and stakeholders’ evaluations in the professional attitude and ethical judgement

|  | 1  Completely  unprepared | 2 | 3 | 4  Undecided | 5 | 6 | 7  Fully prepared |
| --- | --- | --- | --- | --- | --- | --- | --- |
|  | Students%/New graduates%**/Stakeholders**% | | | | | | |
| Respecting patients’ dignity and choices and providing care according to the patient’s needs and culture | 0.0/0.0/**0.0** | 0.0/0.0/**3.6** | 0.0/0.0/**3.6** | 12.0/11.8/**10.7** | 12.0/23.5/**26.7** | 36.0/23.5/**41.1** | 40.0/41.2/**14.3** |
| Recognising and acting within the Dental Board of Australia’s standards and within other professionally relevant laws, ethical guidance and systems | 0.0/0.0/**0.0** | 0.0/0.0/**3.6** | 0.0/5.9/**1.8** | 20.0/11.8/**16.1** | 20.0/11.8/**17.9** | 28.0/29.4/**37.4** | 32.0/41.2/**23.2** |
| Understanding the roles of, and cooperating effectively with, other members of the healthcare team in the best interests of patients | 0.0/0.0/**0.0** | 0.0/0.0/**3.8** | 0.0/7.7/**9.4** | 13.6/0.0/**11.3** | 22.8/7.7/**28.3** | 31.8/38.4/**28.3** | 31.8/46.2/**18.9** |
| Recognising the importance of and demonstrating personal accountability to patients, the regulator, the team and wider community, and putting patients’ interests first and acting as their advocate where appropriate | 0.0/0.0/**0.0** | 0.0/0.0/**1.9** | 4.5/0.0/**7.7** | 9.1/7.1/**19.2** | 18.2/21.4/**19.2** | 45.5/42.9/**40.5** | 22.7/28.6/**11.5** |
| Leading, managing and taking professional responsibility for the actions of colleagues and other members of the team involved in patient care | 0.0/0.0/**2.1** | 4.5/0.0/**6.4** | 0.0/0.0/**8.5** | 13.6/0.0/**23.4** | 31.8/28.6/**29.8** | 36.4/35.7/**23.4** | 13.7/35.7/**6.4** |
| Recognising and complying with local and national systems and processes to support safe patient care, including the safe use of equipment and materials | 0.0/0.0/**0.0** | 0.0/0.0/**3.7** | 9.5/7.1/**3.7** | 9.5/7.1/**14.8** | 23.8/21.4/**25.9** | 28.6/35.7/**38.9** | 28.6/28.7/**13.0** |

* Students (n=28); New graduates (n=18); Stakeholders (n=74)
