# Supplementary material for: Preparedness for practice of newly qualified dental practitioners in the Australian context: an exploratory study
Source: BMC Med Educ. 2022 Aug 18;22:625. doi: 10.1186/s12909-022-03684-1 (PMC9385413; doi:10.1186/s12909-022-03684-1)
Supplement: Supplementary file 5 — Additional file 5: Table 8a. Proportion (%) of students’ and new graduates’ level of self-reported preparedness and stakeholders’ evaluations in the clinical entrepreneurship and financial solvency domain.* [file 12909_2022_3684_MOESM5_ESM.docx]

Table 8a. Proportion (%) of students’ and new graduates’ level of self-reported preparedness and stakeholders’ evaluations in the clinical entrepreneurship and financial solvency domain

|  | 1  Completely  unprepared | 2 | 3 | 4  Undecided | 5 | 6 | 7  Fully prepared |
| --- | --- | --- | --- | --- | --- | --- | --- |
|  | Students%/New graduates%**/Stakeholders**% | | | | | | |
| Understands the characteristics of the health system he or she is employed in and the associated financial pressures and obligations of these systems | 4.5/0.0/**2.1** | 4.5/7.1/**19.1** | 27.3/21.4/**12.8** | 27.3/14.3/**14.9** | 18.2/14.3/**21.3** | 13.7/35.8/**21.3** | 4.5/7.1/**8.5** |
| Being able to explain treatment costs, negotiate with patients around fees and deal with other stakeholders and finances to ensure survival in business | 4.8/0.0/**4.9** | 28.6/14.3/**22.0** | 19.0/14.3/**14.6** | 19.0/7.1/**17.1** | 9.5/42.9/**19.5** | 14.3/14.3/**14.6** | 4.8/7.1/**7.3** |
| Understanding the interface between clinical practice, patient care, and operating a business in conjunction with one's professional and legal obligations as a health professional | 4.5/0.0/**2.1** | 9.1/0.0/**18.8** | 18.2/14.3/**20.8** | 18.2/14.3/**10.4** | 18.2/21.4/**22.9** | 22.7/28.6/**14.6** | 9.1/21.4/**10.4** |

* Students (n=28); New graduates (n=18); Stakeholders (n=74)
