# Supplementary material for: Preparedness for practice of newly qualified dental practitioners in the Australian context: an exploratory study
Source: BMC Med Educ. 2022 Aug 18;22:625. doi: 10.1186/s12909-022-03684-1 (PMC9385413; doi:10.1186/s12909-022-03684-1)
Supplement: Supplementary file 6 — Additional file 6: Table 9a. Proportion (%) of students’ and new graduates’ level of self-reported preparedness and stakeholders’ evaluations in the social and community orientation domain.* [file 12909_2022_3684_MOESM6_ESM.docx]

Table 9a. Proportion (%) of students’ and new graduates’ level of self-reported preparedness and stakeholders’ evaluations in the social and community orientation domain.*

|  | 1  Completely  unprepared | 2 | 3 | 4  Undecided | 5 | 6 | 7  Fully prepared |
| --- | --- | --- | --- | --- | --- | --- | --- |
|  | Students%/New graduates%**/Stakeholders**% | | | | | | |
| Providing culturally safe care that recognises the distinct needs of Aboriginal and Torres Strait Islander Peoples in relation to dental care provision | 0.0/14.3/**2.3** | 9.1/7.1/**4.5** | 13.6/0.0/**6.8** | 31.8/0.0/**18.2** | 13.6/28.6/**27.3** | 13.6/35.7/**29.5** | 18.3/14.3/**11.4** |
| Understanding the current issues relating to inequalities in dental, and how to plan to address these needs | 0.0/0.0/**0.0** | 0.0/14.4/**8.0** | 4.5/21.4/**8.0** | 27.3/7.1/**22.0** | 31.8/21.4/**22.0** | 18.2/14.3/**30.0** | 18.2/21.4/**10.0** |
| Evaluating the impact of social factors on illness, holistically understanding the social situations of one’s patients and their families and/or carers | 0.0/0.0/**0.0** | 0.0/7.1/**5.9** | 0.0/7.1/**9.8** | 18.2/7.1/**23.5** | 45.4/21.4/**29.4** | 18.2/35.9/**21.6** | 18.2/21.4/**9.8** |

* Students (n=28); New graduates (n=18); Stakeholders (n=74)
